# Burden of puerperal sepsis and its associated factors in Ethiopia: a systematic review and meta-analysis

**DOI:** 10.1186/s13690-021-00732-y

**Published:** 2021-11-29

**Authors:** Abenezer Melkie, Enyew Dagnew

**Affiliations:** Department of midwifery, Debre Tabor University, College of Health Sciences, Debre Tabor, Ethiopia

**Keywords:** Burden, Puerperal sepsis, Associated factors, Ethiopia

## Abstract

**Background:**

Puerperal sepsis is a genital tract infection that can occur from amniotic fluid rupture to six weeks after birth. Maternal complication associated with puerperal sepsis includes prolonged hospital stay, septicemia, disseminated intravascular coagulation, pelvic inflammatory disease, infertility, and death. Even though, puerperal sepsis is the fourth leading cause of maternal morbidity and mortality in Ethiopia the overall prevalence of puerperal sepsis and its associated factors are not studied at the national stage. As a result, this systematic review and meta-analysis bring out the pooled prevalence of puerperal sepsis and its associated factors in Ethiopia.

**Methods:**

A variety of data sources such as Pub Med, Web of Science, Science Direct, Embase, Google Scholar, HINARI, and Ethiopian universities online repositories were searched to identify the primary studies which were used for this systematic review and meta-analysis. The article search was conducted from February10/2021-March 10/2021. The quality of the selected primary studies was assessed using the Newcastle - Ottawa quality assessment Scale (NOS). Data extraction was done with Microsoft Excel and then exported to STATA 11 version statistical software for analysis. The Cochran (Q-test) and I2 test statistics were used to assess the heterogeneity of the studies. Publication bias was evaluated by the eggers regression test. Subgroup analysis was performed with region and sample size category.

**Result:**

In this review, a total of 2222 respondents were involved from seven studies. The pooled prevalence of puerperal sepsis was 14.811% (95%CI; 8.46: 21.16; I^2^ = 94.2, *P* ≤ 0.001). Cesarean section delivery (CSD) (OR = 3.26, 95%CI: 1.90, 5.61), membrane rupture≥24 h (OR = 4.04, 95%CI: 2.54, 6.42), being multiparous mother (OR = 3.99, 95%CI: 1.82, 8.78), vaginal examination≥5 times (OR = 3.15, 95%CI: 1.17, 8.52), and anemia (OR = 5.68, 95%CI: 4.38, 7.36) were factors significantly associated with puerperal sepsis.

**Conclusion:**

The prevalence of puerperal sepsis was high in Ethiopia. CSD, membrane rupture≥24 h, being multiparous mother, vaginal examination≥5, and anemia were factors associated with puerperal sepsis. Appropriate standard infection prevention techniques during CSD shall be practiced to reduce the maternal burden of puerperal sepsis. The unnecessary vaginal examination should be discouraged during the intrapartum period. Besides this, routine Iron sulfate supplementation and counsel on iron reach foods during ante partum and postpartum shall be considered for all mothers.

**Supplementary Information:**

The online version contains supplementary material available at 10.1186/s13690-021-00732-y.

## Background

Postpartum endo-mayometrities is a genital tract infection that can strike at any point between the rupture of the membranes or labor and the 42nd postpartum day. Puerperal sepsis is diagnostic if the woman has at least two of the following clinical features: Pelvic pain, Fever (oral T^o^ ≥ 38.5 C^0^), abnormal vaginal discharge, foul odor vaginal discharge, and delay in the involution of the uterus within six weeks of giving birth [1].

Even if maternal mortality is somewhat reducing globally, the majority of maternal deaths occur at the time of giving birth. Puerperal sepsis is avoidable leading cause of maternal morbidity and mortality [2]. Globally; 75,000 maternal deaths occur in a year as a result of puerperal sepsis: among this; 11.6% in Asia, 9.7% in Africa, 7.7% in South America, and 7.7% in Caribbean countries [3, 4]. After postpartum bleeding, unsafe abortion, and pregnancy-induced hypertension, puerperal sepsis is the fourth greatest cause of maternal mortality [5]. Maternal complication associated with puerperal sepsis includes prolonged hospital stay, septicemia, disseminated intravascular coagulation, pelvic inflammatory disease, infertility, and death [6].

Repeated vaginal examination, prolonged labor duration, rupture of membrane for prolonged time, impaired immunity, caesarean section, and retained products of conception are known factors associated with puerperal sepsis [2]. Studies demonstrated that the prevalence of puerperal sepsis were 16.6% in Pakistan [7], 68.65% in India [8], and 72.9% in Sudan [9]. The prevalence of puerperal sepsis varies in different hospitals of Ethiopia which is 5.68% in Dessie referral hospital [10], 8.4% in Addis Abeba black lion specialized hospital [11], 12.9% in Dire Dawa, dil-chora hospital [12], 17.2% in the university of Gondar referral hospital [13], and 33.7% in Bahirdar Felege hiowt referral hospital [14].

In sub Saharan countries including Ethiopia; there is lack of data on puerperal sepsis. In poor resource setting after delivery mothers discharged to their home after short period of follow up and this might not afford enough time to exclude evidence of puerperal sepsis prior to discharge from the health facilities.

Ethiopia has agreed to implement the sustainable development goals (SDGs) to decrease maternal mortality to < 70/100,000 live births through 2030G.C [15]. Thus, in order to carry out this plan and accelerate maternal mortality reduction, it is preferable to identify the causes and contributors to maternal mortality. Having every birth attended by skilled birth attendant, access to comprehensive emergency obstetric care, apply standard infection prevention techniques in the heath facility,and ensure effective referral system are the strategies of reducing maternal morbidity and mortality in relation to sepsis [15].

Knowledge of puerperal sepsis enables to practice the strategies towards this direct causes of maternal mortality and helps to achieve the sustainable developmental goals (SDGs) [15]. However, the effect of puerperal sepsis on maternal morbidity and mortality is minimally understood by different stake holders [15]. Assessing the prevalence of puerperal sepsis and its associated factors at the national level (Ethiopia) is helpful to challenge the fourth leading cause (sepsis) of maternal morbidity and mortality. Despite the fact that some studies have been conducted to assess the prevalence and associated factors of puerperal sepsis, there is a lack of data at the national level to show the prevalence and associated factors of puerperal sepsis.. Moreover, the prevalence and associated factors of puerperal sepsis were incoherent in primary studies. Therefore, this systematic review and meta-analysis aimed to estimate the overall prevalence of puerperal sepsis and its associated factors in Ethiopia.

## Methods

This systematic review and meta-analysis were done by the methodology of preferred reporting items for systematic review and meta-analysis (PRISMA) cheek list (Additional file 1). It was conducted by systematic synthesis of the original studies about puerperal sepsis and associated factors in Ethiopia.

### Searching strategies

International online databases (i.e. Pub Med, Web of Science, Science Direct, Embase, Google scholar, and HINARI), and Ethiopian university’s online repositories were used to search articles. Studies published between October 1, 2000, and January 1, 2021, G. C were included in this systematic review and Meta-analysis. The adapted PECO format was utilized for this systematic review and Meta-analysis. This PICO was comprised of Population (P), Exposure (E), Comparison (C), and Outcome (O) as shown below. In each component of the adapted PECO, search terms are given.

- Population: postpartum mothers
- Exposure: Determinants, associated factors i.e. Caesarean section delivery, ROM≥24 h, being multiparous mother, vaginal examination ≥5, and anemia.
- Comparison(C): Comparison have been adjusted like this: having CSD VS vaginal delivery, those who had ROM≥24 h VS those who had ROM< 24 h, being multiparous mothers primiparous mother, Vaginal examination ≥5 VS vaginal examination < 5 times, and having anemia VS not having anemia.
- Outcome: Prevalence of puerperal sepsis.

Using the above adapted PECO format, the following review questions were created to retrieve as many relevant primary studies as possible:

- Review questions1. What is the national prevalence of puerperal sepsis in Ethiopia?
- Review question 2. What are the factors associated with puerperal sepsis in Ethiopia?

Each database was searched independently with some modifications of the search strategy. Human being and the English language was applied as limiters of the search. The type of searching strategy was line by line and it was done through the title (TI), abstract (Ab), and full-text categories. Boolean operators (“OR”and”AND”) search operator was applied. Synonyms were also utilized to look for more main research. We broadened our search beyond systematic database searches to include retrieving reference lists of papers that met our criteria. In addition, thecited by’ andrelated articles’ capabilities of PubMed were used to conduct further literature searches. A literature search was conducted from February10/2021-March 10/2021.Finally, all studies that matched the title of the review were located and assessed for inclusion criteria. Two independent authors conducted the literature search, with differences handled by discussion and consensus. For PUBMED and Google scholar database searches, a sample of the primary search string has been provided as a supplementary file (Additional file 2).

### Outcome variable measurement

Puerperal sepsis is a genital tract infection that can occur up to six weeks after delivery when the amniotic fluid ruptures, and a woman should have at least two of the following symptoms.: pelvic pain, oral temprature≥38.5 C^0^, abnormal vaginal discharge, foul odours vaginal discharge, and delay in the involution of the uterus [13].

### Independent variable definition

#### Caesarean delivery

Caesarean delivery is defined as the delivery of a fetus through surgical incisions made through the abdominal wall (laparotomy) and the uterine wall (hysterotomy) [16].

#### Rupture of membrane (ROM) ≥24 h

Leakage of amniotic fluid for ≥24 h’s duration of before giving birth.

#### Multiparous mother

Women who give birth ≥2 times after the age of viability regardless of the fetal outcome.

#### Vaginal examination ≥5

When woman have ≥5 times vaginal examination during labor time.

#### Anemia

Hemoglobin level < 11 g/dl for postpartum mother.

### Criteria for inclusion and exclusion

This systematic review and meta-analysis included primary studies of any design that reported magnitude of puerperal sepsis and/or associated factors in Ethiopia. However, primary studies were excluded due to any of the following reasons: (a) no report either prevalence or associated factors of puerperal sepsis, (b) articles without full text, (c) articles with poor quality score, (d) articles whose full text was not available within four weeks of email contact, and (e) narrative reviews, editorials, correspondence, abstracts, and methodological studies. Two authors (AM and ED) evaluated the eligibility of all searched studies independently, and any disagreement were resolved through discussion.

### Study screening and selection

Findings were collected by using online databases and transferred from endnote version six manager to Microsoft excel to remove duplicated articles. There were two stages to the study selection process. First, the title and abstract were screened. Then there was full-text review. The titles and abstracts were checked by two authors independently (AM and ED). Studies that reported the prevalence and/or associated factors of puerperal sepsis were selected for full text review. Following full-text review, any study classified as potentially eligible by either author was considered as a full text and independently screened by both authors.

### Critical appraisal and reliability check

After screening; the quality of the selected primary studies was assessed using the Newcastle - Ottawa quality assessment Scale (NOS) for prevalence and case-control studies. To evaluate cross-sectional studies, we used the following criteria: (1) The sample’s representativeness; (2) Respondents who did not respond; (3) Determination of exposure (risk factor); (4) Based on the study design or analysis, the subjects in different outcome groups are comparable. Confounding variables are kept under control; (5) .Evaluation of the outcome; (6) Statistical test When articles received 7 points out of 9 for cross-sectional research, they were considered high-quality. We used the following criteria to appraisal case-control studies: (1) adequate case definition; (2) representativeness of the cases; (3) control selection; (4) control definition; (5) comparability of cases and controls based on design or analysis; (6) exposure determination; (7) Cases and controls were exposed using the same method; (8) the non-response rate was calculated. When articles scored 9 points out of 10 for case-control studies, they were considered high-quality. Two writers (AM and ED) scored the data, with disagreements handled by discussion and consensus. The quality scores that calculated the level of agreement for the independent reviews are reported (Additional file 3). During the quality assessment of each primary study, much stress was given to the suitability of the study aims, study design, sampling technique, data collection technique, statistical analysis, any sources of bias, and its management technique.

### Data extraction

After collecting studies by using different online databases (AM) &(ED), autonomously extracted all required data with a standardized data extraction tool. We used two criteria for variables that are used for data extraction [1]. Clear and steady measurement of the variable in the included primary studies [2]. Statistically significant variable in the primary studies which is showed by AOR. The first author’s name, year of publication, region, research area, study design, sample size, prevalence, and study quality were among the variables on which data was retrieved from the assessed primary studies. These crucial data were extracted utilizing a data extraction format created on a Microsoft Excel spreadsheet that was initially validated by extracting sample data from some suitable articles before making significant changes for the real data extraction. During extraction, we did not face inconsistent reporting of data for variables in the incorporated primary studies but if we got this inconsistent we would have used data transformation.

### Data analysis

Data were entered into Microsoft Excel and then exported to the statistical software STATA Version 11 for analysis. The *p*-values of I^2^ statistics were used to assess heterogeneity in reported prevalence. Random-effect model were used if heterogeneity within the studies observed. Fixed-effect model were used if homogeneity within the studies seen. While assessing the polled prevalence of puerperal sepsis in Ethiopia, heterogeneity was observed between studies (I^2^ = 94.2%, *P* > 0.01). To assess the pooled prevalence of puerperal sepsis in Ethiopia, random-effect models were used. Subgroup analysis was done to identify possible source heterogeneity by using sample size categories. Eggers regression test was used to assess publication bias between the studies.

## Result

### Search result

**Fig. 1:**
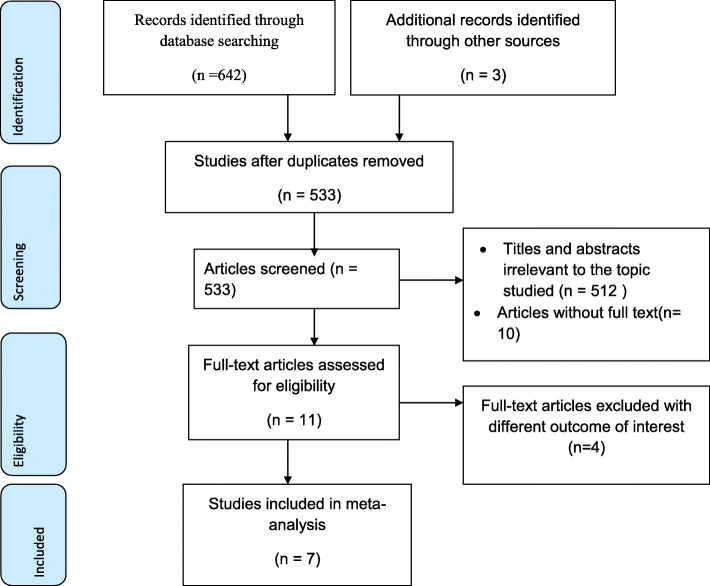
flow chart for systematic review and meta-analysis

645 articles were retrieved using a search strategy regarding the prevalence and associated factors of puerperal sepsis in Ethiopia at **Pub Med, Web of Science, Science Direct, Embase, Google scholar and HINARI, and Ethiopian universities online repository**. All articles found during the search were exported to Endnote, and 112 were removed due to duplication. Five hundred thirty-three studies were screened for eligibility, relevance, accessibility, and outcome of interest. Accordingly, 512 studies were excluded due to irrelevant titles, and 10 studies due to inaccessible full text. Five articles were removed due to different outcomes of interest. Finally, 6 studies were included in this systematic review and meta-analysis (Fig. 1).

### Study characteristics

A total of seven studies with a total of 2222 sample sizes were included in this systematic review and meta-analysis. Based on study design; five studies were cross-sectional [10–14] and two were case-control studies [1, 17]. In this study, two regions and two administrative cities were included. Three studies were from the Amhara region [10, 13, 14], two study from the Oromia region [1, 17], one study from Addis Ababa city [11], and one study from Dire Dawa city [12] (Table 1).

**Table 1:** Descriptive summary of seven studies included in the meta-analysis of the prevalence of puerperal sepsis and its associated factors in Ethiopia

Author	Publication year	Region	study area	study design	sample size	Prevalence (%)	Quality Of the study
**Daniel eta.l**	2019	Amhara	Diredawa	cross-sectional	441	12.9252	10
**Alemale etal.**	2018	Amhara	Felege hiwot referral hospital	cross-sectional	166	33.735	10
**Getu etal**	2019	Oromia	Public Hospitals in west shewa	Case-control	280	–	10
**Fikremelkot Temesgen**	2014	Addis Abeba	Black lion hospital	cross-sectional	394	8.3756	9
**Daniel** **Etal**	2019	Amhara	University of Gondar referral hospital	Cross-sectional	219	17.2	10
**Nigussie Abebaw**	2018	Amhara	Dessie referral hospital	cross-sectional	422	5.6872	9
**Hana Liben**	2016	Oromia	Jimma specialized hospital	Case-control	300	–	9

### Publication bias

**Fig. 2:**
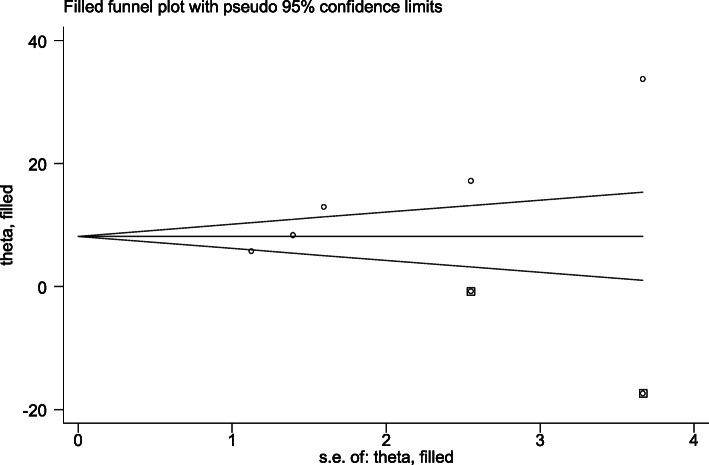
Funnel plot for the prevalence of puerperal sepsis and its associated factors after publication bias was adjusted by trim fill analysis

The egger’s regression test was used to evaluate the likelihood of publication bias within the studies [18, 19]. The test result revealed that publication bias was seen between the studies (egger’s regression test *p*-value = 0.006). To eliminate publication bias across the studies, Duval and Tweedie’s Trim and Fill analyses were used. The adjusted pooled prevalence of puerperal sepsis after adding two studies with fill and trim analysis was 8.539%(1.691,15.388). Hence, publication bias was solved when two studies were incorporated in the funnel plot by trim fill analysis (Fig. 2).

### Prevalence of puerperal sepsis

**Fig. 3:**
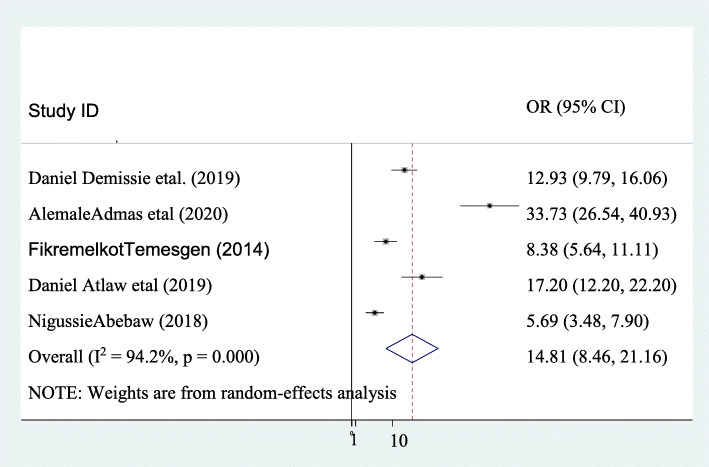
Pooled prevalence of puerperal sepsis and its associated factors in Ethiopia

The pooled prevalence of puerperal sepsis in Ethiopia was 14.811% (95%CI; 8.46: 21.16; I^2^ = 94.2, *P* ≤ 0.001). This systematic review and meta-analysis revealed that marked heterogeneity was seen within the included studies (I^2^ = 94.2, *P* ≤ 0.001). Therefore, random-effect models were applied to measure the pooled prevalence of puerperal sepsis in Ethiopia. In the included studies the maximum prevalence of puerperal sepsis was reported by Alemale etal. Which was 33.73% [14] and the lowest prevalence of puerperal sepsis was reported by Nigusie Abebaw which was 5.687% [10] (Fig. 3).

### Subgroup analysis

Subgroup analysis was conducted to detect the potential source of heterogeneity within the included studies for this systematic review and meta-analysis. Subgroup analysis was performed with sample size category and by the region where the studies were done. Based on the sample size category, the maximum prevalence of puerperal sepsis was observed in studies that had a sample size of < 300 with 25.26% (95CI:9.06, 41.46)**.** According to the region, the highest prevalence of puerperal sepsis was seen in the Amhara region with a prevalence of 18.54% (95CI:3.69, 33.39) (Table 2).

**Table 2:** Subgroup analysis of the prevalence of puerperal sepsis in Ethiopia

Variables	Characteristics	Included primary studies	Sample size	Prevalence
With region	Amhara	3	807	18.54% (95CI:3.69, 33.39)
	Self-administrative town	2	835	10.58(95CI:6.13,15.04)
With sample size	< 300	2	385	25.26% (95CI:9.06, 41.46)
	≥300	3	1257	8.88(95CI:4.83,12.92)

### Factors associated with puerperal sepsis

CSD 3.26(1.90, 5.61), membrane rupture≥24 h 4.04(2.54, 6.42), being multiparous mother 3.99(1.82, 8.78), vaginal examination≥5, 3.15(1.17, 8.52), and anemia 5.68(95CI:4.38, 7.36) were factors associated with puerperal sepsis in this systematic review and meta-analysis.

### The association between puerperal sepsis and CSD

**Fig. 4:**
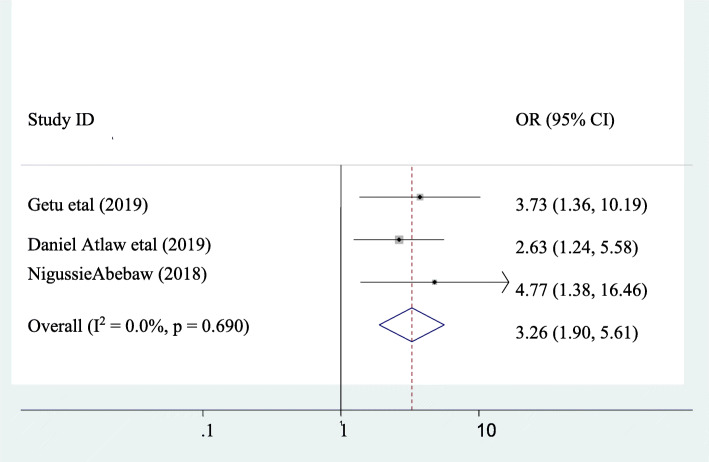
pooled odds ratio of the association between C/S delivery and puerperal sepsis

Three studies were incorporated in this class of systematic review and meta-analysis [1, 10, 13]. A significant association was observed between puerperal sepsis and CSD. The odds of developing puerperal sepsis among women who had CSD were 3.26 times higher than as compared to those who had a vaginal delivery (AOR =3.26, 95% CI: 1.90, 5.61). Heterogeneity within the studies was not observed (I^2^ = 0). Hence, a fixed-effect model was applied to verify the association (Fig. 4).

### The association between puerperal sepsis and membrane rupture ≥24 h

**Fig. 5:**
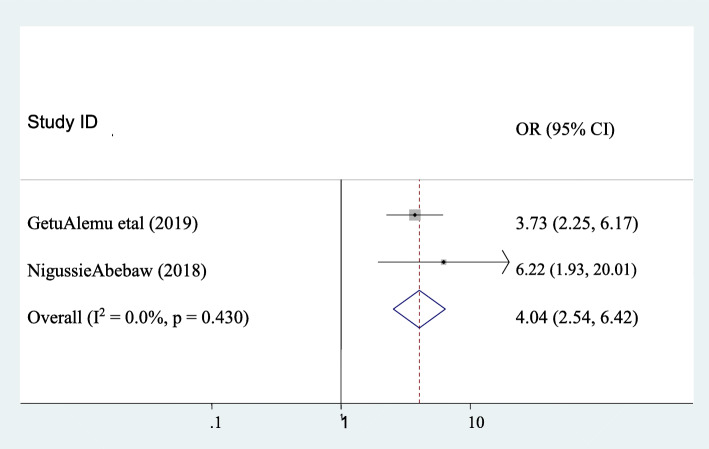
Pooled odds ratio of the association between membrane rupture≥24 h and puerperal sepsis

The association between puerperal sepsis and membrane rupture was assessed by using two studies [1, 10]. The finding showed that a significant association was observed between puerperal sepsis and membrane rupture≥24 h. Accordingly, the likelihood of puerperal sepsis was 4.04 times higher in those mothers who had membrane rupture≥24 h as compared to their counterparts (AOR: 4.04, 95CI:2.54, 6.42). Heterogeneity was not observed across the studies (I^2^ = 0). Hence, a fixed-effect model was applied to determine the association (Fig. 5).

### The association between puerperal sepsis and being multiparous mother

**Fig. 6:**
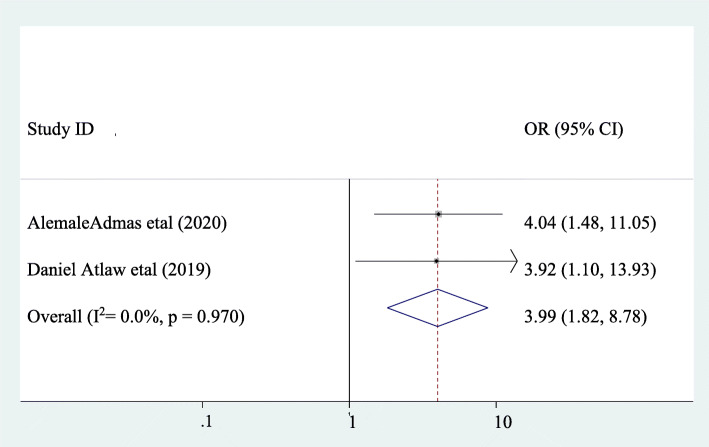
Pooled odds ratio of the association between being multiparous mother and puerperal sepsis

The association between puerperal sepsis and multiparous mother was assessed by using two studies [13, 14]. A significant association was observed between puerperal sepsis and being a multiparous mother. Accordingly, the likelihood of puerperal sepsis was 3.99 times higher in multiparous mothers as compared to their counterparts (AOR: 3.99, 95CI:1.82, 8.78). Heterogeneity was not observed within the studies (I^2^ = 0). Hence, a fixed-effect the model was applied find out the association (Fig. 6).

### The association between puerperal sepsis and having vaginal examination ≥5 times

**Fig. 7:**
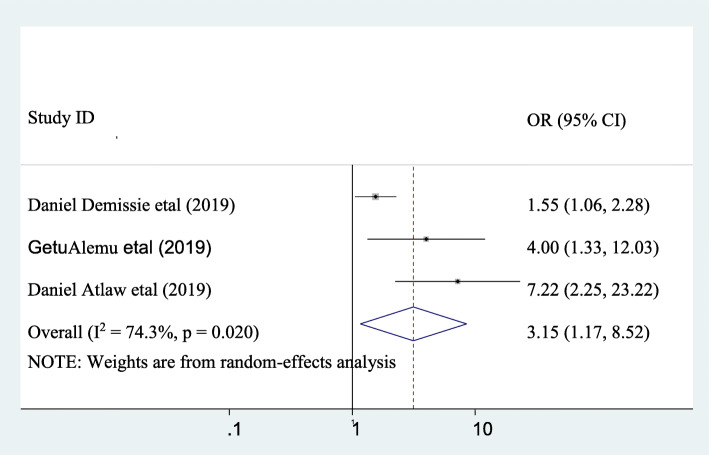
pooled odds ratio of the association between having vaginal examination ≥5 and puerperal sepsis

The association between puerperal sepsis and vaginal examination ≥5 were assessed by using three studies [1, 12, 13]. The finding revealed that significant association was seen between puerperal sepsis and vaginal examination ≥5 times. Accordingly, the likelihood of puerperal sepsis was 3.15 times higher in those mothers who had vaginal examination ≥5 times as compared to their counterpart (AOR: 3.15, 95CI, 1.17, 8.52). Moderate heterogeneity across the studies was observed (I^2^ = 74.3). Hence, random- effect the model was applied to find out the association (Fig. 7).

### The association between puerperal sepsis and anemia

**Fig. 8:**
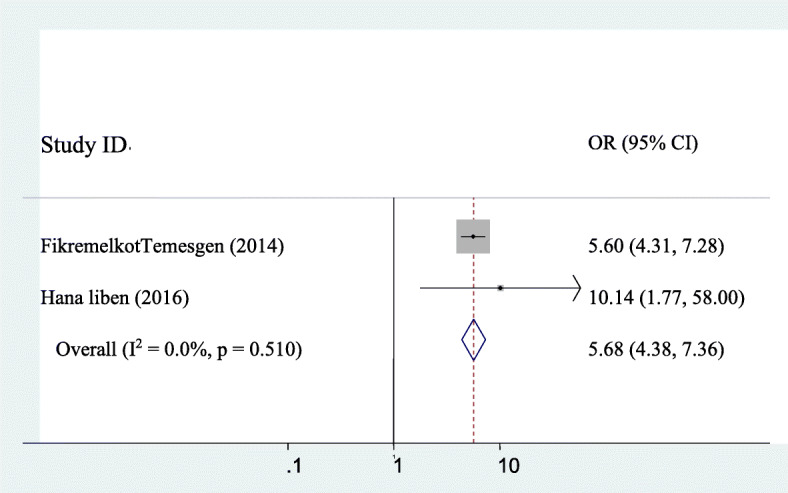
pooled odds ratio of the association between anemia and puerperal sepsis

The association between puerperal sepsis and anemia were assessed by using two studies [11, 20]. Significant association was observed between puerperal sepsis and anemia. Accordingly, the likelihood of puerperal sepsis was 5.68 times higher in those mothers who had anemia as compared to their counterpart (AOR: 5.68, 95CI:4.38, 7.36). Heterogeneity across the studies was not seen (I^2^ = 0). Hence, fixed-effect the model was applied to determine the association (Fig. 8).

## Discussion

This systematic review and meta-analysis was aimed to assess the pooled prevalence of puerperal sepsis and associated factors in Ethiopia. This study has showed that the overall prevalence of puerperal sepsis in Ethiopia was 14.811%(95%CI: 8.46, 21.16). The prevalence of puerperal sepsis in the present study is in line with the studies done in Lahore Pakistan (16.6%) [7],Nigeria 11.4% [21]. The finding of the present study was higher than a study done in Osun State, Nigeria (0.78%) [6], and Sindh Pakistan (3.89%) [22] but lower than studies done in Sudan (72.9%) [9],Zambia (34.8%) [23], and India (68.65%) [8]. The difference could be the variation of sample size, Level of hygiene at the community and individual level, study area, causative agent for puerperal sepsis, institutional delivery coverage, and quality of maternity service. Good quality of maternity service and high rate of institutional delivery coverage decreases the rates of puerperal sepsis. Beside to this, good hygiene of the community and maternity women at an individual level might be helpful to prevent puerperal sepsis.

The subgroup analysis of this study showed that the prevalence of puerperal sepsis among postpartum mothers was significantly varies across regions. The highest prevalence of puerperal sepsis was observed in Amhara region 18.54% (95CI:3.69, 33.39) and the lowest prevalence was seen in Self-administered cities (Addis Abeba and Dire Dawa) 10.58(95CI:6.13, 15.04). This could be the difference of awareness for maternal and child health services, distance of health institution which gives maternity services from their home, and availability health institutions and medical equipments. The awareness of postpartum mothers who lives in the town administrative (Addis Abeba and Dire Dawa) about the availability of maternity health services is high due to their exposure for radio, television, and mass media, and press. Beside to this, postpartum mothers who live in the town administrative (Addis Abeba and Dire Dawa) have good infrastructures such as road, and ambulance to rich timely and get maternity services in compare to who lives in Amhara region (Almost all part is rural and distant to get maternity health services).

The current study revealed that CSD was significantly associated with puerperal sepsis. The odds of developing puerperal sepsis among women who had CSD were 3.26 times higher than as compared to those who had a vaginal delivery (AOR =3.26, 95% CI: 1.90, 5.61). These finding supported by studies done in Scotland [24], and Tanzania [25] which was reported that having CSD was associated with puerperal sepsis. The possible explanation could be due to poor infection prevention technique during operation procedure, Loss significant amount of blood due to poor homeostasis during CSD, and High load of obstetric of cases in the maternity ward. Ethiopia is one of the countries which have a high population with poor health service coverage especially maternal and child health service which might lead puerperal sepsis.

Membrane rupture≥24 h was significantly associated with puerperal sepsis. Accordingly, the likelihood of puerperal sepsis was 4.04 times higher in those mothers who had membrane rupture≥24 h as compared to their counterparts (AOR: 4.04, 95CI:2.54, 6.42). This result is similar with studies done in Pakistan [22], and Kenya [26] which reported that membrane rupture ≥24 h is associated with puerperal sepsis. This could be once the membrane (which gives protection for upper reproductive tract from ascending infection) ruptured bacterial organisms which arises from external environment and vagina have got the chance to go through dilated cervix to the upper internal reproductive tract. Moreover, if a woman comes with ruptured membrane with dilated cervix and took long time to give birth the number of vaginal examination increased as time gooses and this might leads to puerperal sepsis.

Being multiparous mother was significantly associated with puerperal sepsis. The likelihood of puerperal sepsis was 3.99 times higher in multiparous mothers as compared to their counterparts (AOR: 3.99, 95CI:1.82, 8.78). This finding is similar with the studies done in United States of America [27], and Pakistan [22] which reported that being multiparous mother is associated with puerperal sepsis. This could be due to when parity increases some obstetrical complications such as uterine rupture, Post partum hemorrhage, and retained placenta increases and this complication also increases the rate of puerperal sepsis due to numerous manipulation of the internal reproductive tract of the mother.

Having vaginal examination ≥5 times was significantly associated with puerperal sepsis. Accordingly, the likelihood of puerperal sepsis was 3.15 times higher in those mothers who had vaginal examination ≥5 times as compared to their counterpart (AOR: 3.15, 95CI, 1.17, 8.52). This result is similar with the studies done in Kenya [28] Egypt [29], South Asia [30]. This could be when mothers have unnecessary multiple vaginal examination during labor and delivery we inoculate bacteria from outside environment and vagina to the internal reproductive organs and this could leads to puerperal sepsis.

The present study showed that anemia was significantly associated with puerperal sepsis. The likelihood of puerperal sepsis was 5.68 times higher in those mothers who had anemia as compared to their counterpart (AOR: 5.68, 95CI:4.38, 7.36). This study is similar to the study done in Scotland [24], and Nigeria [21]. The possible explanation could be anemic mothers lack natural tolerance for infection and this might leads to puerperal sepsis. Ethiopia is one of the countries which have poor socio-economic status; due to this pregnant and postpartum mothers face a lack of a balanced diet to challenge anemia and its associated complications like sepsis.

### Limitation

This study is limited by the identifying of a few numbers of studies reporting puerperal sepsis, of which merely seven were deemed to be good procedural quality. This study might not represent the overall prevalence of puerperal sepsis at the country level due to the small number of studies and lack of representations of the different regions in Ethiopia.

## Conclusion

The prevalence of puerperal sepsis was high in Ethiopia. CSD, Membrane rupture≥24 h, Being a multiparous mother, vaginal examination≥5, and anemia were factors significantly associated with puerperal sepsis. Appropriate standard infection prevention techniques during CSD shall be practiced to reduce the maternal burden of puerperal sepsis. The frequent vaginal examination should be discouraged during the intrapartum period. Besides this, routine Iron sulfate supplementation and counsel on iron reach foods during antepartum and postpartum shall be considered for all mothers.

## Supplementary Information

**Additional file 1.** PRISMA 2009 Checklist

**Additional file 2.**

**Additional file 3.** Table 1 Quality of assessment of articlesusing Newcastle - Ottawa quality assessment Scale (NOS): (Adapted for cross-sectional studies)

## Data Availability

Data will be obtainable on request from the principal investigator.

## Abbreviations

OR: Odds ratio
CI: Confidence interval
CSD: Cesarean section delivery
SDGs: Sustainable developmental goals
