# Supplementary material for: Burden of puerperal sepsis and its associated factors in Ethiopia: a systematic review and meta-analysis
Source: Arch Public Health. 2021 Nov 29;79:216. doi: 10.1186/s13690-021-00732-y (PMC8628469; doi:10.1186/s13690-021-00732-y)
Supplement: Supplementary file 2 — Additional file 2. [file 13690_2021_732_MOESM2_ESM.docx]

| Search | Query | Search results* |
| --- | --- | --- |
| 1.Google scholar | "Prevalence" , "magnitude" , "proportion" , "puerperal sepsis" , "associated factors" , "determinants" ,"Ethiopia". Searching terms were established using the Boolean operator “and” and “or” | 57 |
| 2. PubMed | ("epidemiology"[Subheading] OR "epidemiology"[All Fields] OR "prevalence"[All Fields] OR "prevalence"[MeSH Terms] OR "magnitude"[All Fields]) AND puerperal [All Fields] AND sepsis [All Fields] AND associated [All Fields] AND factors [All Fields] AND ("Ethiopia"[MeSH Terms] OR "Ethiopia"[All Fields]) | 473 |
| 3. Other data bases | Web of Science, Science direct, Embase, HINARI, and Ethiopian universities online repository | 115 |
| 4. Total search |  | 645 |
| 5. Number of candidate for inclusion |  | 12 |
| 6. Excluded with reason |  | 5 |
| 7. Studies included in this review |  | 7 |

Date of search: February10/2021-March 10/2021
