# Supplementary material for: Burden of puerperal sepsis and its associated factors in Ethiopia: a systematic review and meta-analysis
Source: Arch Public Health. 2021 Nov 29;79:216. doi: 10.1186/s13690-021-00732-y (PMC8628469; doi:10.1186/s13690-021-00732-y)
Supplement: Supplementary file 3 — Additional file 3. Table 1 Quality of assessment of articlesusing Newcastle - Ottawa quality assessment Scale (NOS): (Adapted for cross-sectional studies) [file 13690_2021_732_MOESM3_ESM.docx]

**Table 1: Quality of assessment of articlesusing Newcastle - Ottawa quality assessment Scale (NOS): (Adapted for cross-sectional studies)**

| Studies | **Selection** | | | | **Comparability** | **Outcome** | | **Total score** |
| --- | --- | --- | --- | --- | --- | --- | --- | --- |
|  | Representativeness  (1) | Sample size  (1) | Non-respondents  (1) | Ascertainment of the exposure (risk factor)  (2) | The subjects in different outcome groups are comparable, based on the study design or analysis. Confounding factors are controlled (2) | Assessment of the outcome  (2) | Statistical test  (1) |  |
| **Daniel etal.** | 1 | 1 | 1 | 2 | 2 | 2 | 1 | 10 |
| **Alemale etal.** | 1 | 1 | 1 | 2 | 2 | 2 | 1 | 10 |
| **Fikremelkot Temesgen** | 1 | 1 | 1 | 2 | 2 | 1 | 1 | 9 |
| **Daniel etal.** | 1 | 1 | 1 | 2 | 2 | 2 | 1 | 10 |
| **Nigussie Abebaw** | 1 | 1 | 1 | 2 | 2 | 2 | 1 | 9 |

**Table 1: Quality of assessment of articles using Newcastle - Ottawa quality assessment Scale (NOS)Adapted for case control studies**

| Studies | **Selection** | | | | **Comparability** | **Exposure** | | | **Total score** |
| --- | --- | --- | --- | --- | --- | --- | --- | --- | --- |
|  | Representativeness of the cases  (1) | Is the case definition adequate  (1) | Selection of Controls  (1) | Definition of Controls (1) | Comparability of cases and controls on the basis of the design or analysis (2) | Ascertainment of exposure  (2) | Same method of ascertainment for cases and controls  (1) | Non-Response rate  (1) |  |
| **Getu etal.** | 1 | 1 | 1 | 1 | 2 | 2 | 1 | 1 | 10 |
| **Hana Liben** | 1 | 1 | 1 | 1 | 1 | 2 | 1 | 1 | 9 |
